# Conservation resource allocation, small population resiliency, and the fallacy of conservation triage

**DOI:** 10.1111/cobi.13696

**Published:** 2021-05-04

**Authors:** David A. Wiedenfeld, Allison C. Alberts, Ariadne Angulo, Elizabeth L. Bennett, Onnie Byers, Topiltzin Contreras‐MacBeath, Gláucia Drummond, Gustavo A. B. da Fonseca, Claude Gascon, Ian Harrison, Nicolas Heard, Axel Hochkirch, William Konstant, Penny F. Langhammer, Olivier Langrand, Frederic Launay, Daniel J. Lebbin, Susan Lieberman, Barney Long, Zhi Lu, Michael Maunder, Russell A. Mittermeier, Sanjay Molur, Razan Khalifa al Mubarak, Michael J. Parr, Jonah Ratsimbazafy, Anders G. J. Rhodin, Anthony B. Rylands, Jim Sanderson, Wes Sechrest, Pritpal Soorae, Jatna Supriatna, Amy Upgren, Jean‐Christophe Vié, Li Zhang

**Affiliations:** ^1^ American Bird Conservancy The Plains VA 20198 U.S.A.; ^2^ Ecoleaders 4705 Park Blvd., Suite 102–126 San Diego CA 92103 U.S.A.; ^3^ IUCN SSC Amphibian Specialist Group 3701 Lake Shore Blvd. W, P.O. Box 48586 Toronto ON M8W 1P5 Canada; ^4^ Wildlife Conservation Society 2300 Southern Blvd. Bronx NY 10460 U.S.A.; ^5^ IUCN SSC Conservation Planning Specialist Group 12101 Johnny Cake Ridge Road Apple Valley MN 55124 U.S.A.; ^6^ Centro de Investigaciones Biológicas Universidad Autónoma del Estado de Morelos Avenida Universidad 1001, Col. Chamilpa, CP 62209 Cuernavaca Morelos Mexico; ^7^ Fundação Biodiversitas Avenida Celso Porfírio Machado No. 1813, Belvedere Belo Horizonte MG 30320–400 Brazil; ^8^ The Global Environment Facility 1818 H Street NW Rm N8‐800 Washington DC 20433 U.S.A.; ^9^ Conservation International Arlington VA 22202 U.S.A.; ^10^ Mohamed bin Zayed Species Conservation Fund P.O. Box 13112 Abu Dhabi UAE; ^11^ Department of Biogeography and IUCN SSC Invertebrate Conservation Committee Trier University Trier 54286 Germany; ^12^ Margot Marsh Biodiversity Foundation 403 Poplar Road Flourtown PA 19031 U.S.A.; ^13^ Global Wildlife Conservation Austin TX 78704 U.S.A.; ^14^ Critical Ecosystem Partnership Fund Arlington VA 22202 U.S.A.; ^15^ Panthera New York NY 10018 U.S.A.; ^16^ Center for Nature and Society, School of Life Sciences Peking University Beijing 100871 China; ^17^ Center for Ecology and Conservation University of Exeter Penryn Campus Cornwall TR10 9FE U.K.; ^18^ Zoo Outreach Organization 12 Thiruvannamalai Nagar, Saravanampatti – Kalapatti Road, Saravanampatti Coimbatore Tamil Nadu 641 035 India; ^19^ Environment Agency ‐ Abu Dhabi P.O. Box 45553 Abu Dhabi UAE; ^20^ Groupe d'Etude et de Recherche sur les Primates de Madagascar Antananarivo Madagascar; ^21^ Chelonian Research Foundation Arlington VT 05250 U.S.A.; ^22^ Department of Biology FMIPA, University of Indonesia Depok 16421 Indonesia; ^23^ Fondation Franklinia Place de la Fusterie 5 Geneva 1204 Switzerland; ^24^ Key Laboratory for Biodiversity Science and Ecological Engineering, Ministry of Education Institute of Ecology Beijing Normal University Beijing 100875 China

**Keywords:** conservation triage, extinction, financial resources, small populations, zero extinction

## Abstract

Some conservation prioritization methods are based on the assumption that conservation needs overwhelm current resources and not all species can be conserved; therefore, a conservation triage scheme (i.e., when the system is overwhelmed, species should be divided into three groups based on likelihood of survival, and efforts should be focused on those species in the group with the best survival prospects and reduced or denied to those in the group with no survival prospects and to those in the group not needing special efforts for their conservation) is necessary to guide resource allocation. We argue that this decision‐making strategy is not appropriate because resources are not as limited as often assumed, and it is not evident that there are species that cannot be conserved. Small population size alone, for example, does not doom a species to extinction; plants, reptiles, birds, and mammals offer examples. Although resources dedicated to conserving all threatened species are insufficient at present, the world's economic resources are vast, and greater resources could be dedicated toward species conservation. The political framework for species conservation has improved, with initiatives such as the UN Sustainable Development Goals and other international agreements, funding mechanisms such as The Global Environment Facility, and the rise of many nongovernmental organizations with nimble, rapid‐response small grants programs. For a prioritization system to allow no extinctions, zero extinctions must be an explicit goal of the system. Extinction is not inevitable, and should not be acceptable. A goal of no human‐induced extinctions is imperative given the irreversibility of species loss.

## Abstract 2

*Article impact statement*: Conservation triage is not necessary for species conservation. Adequate resources exist; even species with small populations can recover.

## Abstract (es)

Asignación de Recursos para la Conservación, Resiliencia de Poblaciones Pequeñas y la Falacia del Triaje de Conservación

**Resumen:**

Algunos métodos de priorización de la conservación están basados en el supuesto de que las necesidades de la conservación superan a los actuales recursos y que no todas las especies pueden ser conservadas; por lo tanto, se necesita un esquema de triaje (esto es, cuando el sistema está abrumado, las especies deben dividirse en tres grupos con base en su probabilidad de supervivencia y los esfuerzos deben enfocarse en aquellas especies dentro del grupo con las mejores probabilidades de supervivencia y a aquellas en el grupo sin probabilidades de supervivencia o aquellas en el grupo que no necesita esfuerzos especializados para su conservación se les deben reducir o negar los esfuerzos de conservación) para dirigir la asignación de recursos. Discutimos que esta estrategia para la toma de decisiones no es apropiada porque los recursos no están tan limitados como se asume con frecuencia y tampoco es evidente que existan especies que no puedan ser conservadas. Por ejemplo, tan sólo un tamaño poblacional pequeño no es suficiente para condenar a una especie a la extinción; contamos con ejemplos en plantas, reptiles, aves y mamíferos. Aunque actualmente todos los recursos dedicados a la conservación de todas las especies amenazadas son insuficientes, los recursos económicos mundiales son vastos y se podrían dedicar mayores recursos a la conservación de especies. El marco de trabajo político para la conservación de especies ha mejorado, con iniciativas como los Objetivos de Desarrollo Sustentable de la ONU y otros acuerdos internacionales, el financiamiento de mecanismos como el Fondo para el Medio Ambiente Mundial, y el surgimiento de muchas organizaciones no gubernamentales mediante programas de subsidios pequeños hábiles y de respuesta rápida. Para que un sistema de priorización no permita las extinciones, las cero extinciones deben ser un objetivo explícito del sistema. La extinción no es inevitable y no debería ser aceptable. El objetivo de cero extinciones inducidas por humanos es imperativo dada la irreversibilidad de la pérdida de especies.

## Introduction

Although Earth is inarguably facing a sixth species extinction wave induced by human impacts on the planet's ecosystems, different views exist concerning its inevitability and pace. To most effectively mitigate what all specialists agree would be a catastrophic loss of biological diversity (Secretariat of the Convention on Biological Diversity 2001; Intergovernmental Science‐Policy Platform on Biodiversity and Ecosystem Services 2019), it is necessary to better understand the threats to species survival, the availability and effectiveness of mitigation options, and the relative costs of action or inaction.

Starting in the early years of Western conservation more than a century ago, thoughtful scientists began considering how to make the best use of limited available resources (Cokinos 2000; Van Dyke 2008). This led to many and varied prioritization schemes for allocating resources, including systems aimed at saving species with better survival prospects, while not wasting precious resources on those considered likely to be doomed despite best efforts. These follow the concepts of human medical triage, a topic that has suddenly become highly relevant again with the COVID‐19 pandemic overwhelming medical capacity in some areas (e.g., Maves et al. 2020). In recent years, these latter conservation prioritization schemes have become known as “conservation triage” or “environmental triage” (Lovejoy 1976; Cornwall 2018). Concepts of conservation triage have generated considerable discussion (Hayward & Castley 2018).

The ethical underpinnings of triage in species conservation have been substantially debated (e.g., Wilson & Law 2016; Vucetich et al. 2017). The emergence of new social and political movements emphasizing the need to halt extinction shows that for many conservationists, triage is an unacceptable approach. Humans assign high values to the various elements of nature and their conservation, for maintaining biological diversity, and avoiding the extinction of any species. The ethical considerations clearly point to avoiding extinctions. Besides this ethical axis, however, evaluation of conservation triage must consider its practical component.

The underlying premise of conservation triage is that when resources are limited and the system for addressing conservation problems is overwhelmed, species should be divided into three groups based on likelihood of survival, and efforts should be focused on those species in the group with the best survival prospects and reduced or denied to those in the group with no survival prospects and to those in the group not needing special efforts for their conservation (Noss 1996). Recent authors have shied away from the use of the word *triage* because of its connotations of allowing species to go extinct, preferring to use *prioritization* (Cornwall 2018), and have developed mathematical tools for assisting prioritization decisions (Joseph et al. 2009; Gerber et al. 2018). Use of such tools may be couched in terms of “smart decision making” and “wise resource allocation” (Bottrill et al. 2008) or avoiding “misallocation of scarce conservation resources” (Joseph et al. 2009), but the result is nonetheless that species are assigned values based on the economics of their recovery, and those deemed uneconomic to save are assigned fewer resources for their recovery (Jachowski & Kesler 2008). Although none of these prioritization systems explicitly identify species to be allowed to go extinct, the effect of diverting resources away from uneconomic recovery plans accepts that some species may be “left behind,” that is, provided no resources for their conservation (Gerber et al. 2018). Three examples in peer‐reviewed literature include Wiens et al. (2012), who state that “[w]e must…accept that conservation triage may sometimes be necessary”; Hebblewhite (2017), who argues that “triage may be required through revising the idealistic goals of conserving all [populations] of Canada's woodland caribou” *Rangifer tarandus caribou*; and Cornwall (2018), who reports that “some scientists” argue that “[t]o do the greatest good…, governments need to consider shifting resources from endangered species and populations that are getting too much attention to those not getting enough. That could mean resolving not to spend money on some species for which the chance of success appears low, such as the vaquita, [*Phocoena sinus*].” In popular literature, Chris Packham, a well‐known naturalist in the United Kingdom, stated that “we won't be able to save it all, so let's do the best we can” and “[i]t may well be that we can lose the cherries from the cake. … Save the Kalahari: that would be better” (Benedictus 2009). In private conversations with some of us (T.C.‐M., S.L., B.L., R.M., and J.‐C.V.), financial donors and government officials have expressed that species such as vaquita, saola (*Pseudoryx nghetinhensis*), northern white rhinoceros (*Ceratotherium simum cottoni*), and Sumatran rhinoceros (*Dicerorhinus sumatrensis*) cannot be recovered and therefore donors or officials will not support their conservation. All these examples represent conservation triage.

We do not argue against prioritization in use of resources, but rather argue that conservation triage is neither appropriate nor needed and that no extinctions must be an explicit goal of conservation. We argue this because resources (financial, human, and time) are not as limited as is often assumed and are not a zero‐sum game, and the number of extant species that cannot be recovered to a global population at greatly reduced risk of extinction is very low.

## Species Conservation Needs and Available Resources

Currently, adequate resources are not readily available for conservation, leading some to propose a conservation triage approach in which species, such as the vaquita, would be allowed to go extinct (Cornwall 2018). However, although conservationists may need to adjust funding priorities to make the most efficient use of resources, getting to the point of abandoning some species and allowing them to go extinct is not a necessity. The questions therefore should be what are the actual needs for conservation resources to address all species conservation and can conservationists expand the resource base to cover all these needs?

Current financial investment in global conservation in the context of the current development model is clearly inadequate to maintain all populations of all species at present levels (James et al. 2001; McCarthy et al. 2012; Waldron et al. 2013). Estimates for the current global conservation expenditure are difficult to come by and are by nature subjective and broad. For the late 1990s, global spending on management of existing protected areas was estimated at US$6 billion (James et al. 2001). A decade later, an estimated US$21.5 billion was being spent annually on major conservation programs, including management of existing conservation areas and unprotected‐area‐specific conservation (Waldron et al. 2013). However, these values are estimates of actual spending at the times those analyses were conducted. Beyond this, the funding necessary to adequately conserve species was estimated to be US$76.1 billion per year (McCarthy et al. 2012), more than 3 times what is actually spent (Waldron et al. 2013). Yet overall, resources for all sectors of society are hardly scarce. The estimated value of the global economy for 2018 was about US$82.5 trillion (World Bank 2020), an amount more than 1000 times larger than the estimated need for conservation in 2012 (McCarthy et al. 2012).

Of course, achieving even a doubling of conservation funding would be a daunting task, although not impossible. Aside from only avoiding imminent extinctions, a functional planet will require a reordering of how humanity views economic development and human welfare, including applying assets to biodiversity conservation. The effects of the COVID‐19 pandemic on the human world and economics are certainly challenging governmental and nongovernmental resources, but at the same time provide opportunities. Recovery strategies in a post‐COVID‐19 world can be optimized to reboot economies and benefit biodiversity conservation (Pearson et al. 2020).

Some governments are already committed. At the 2010 Meeting of the Conference of the Parties to the Convention on Biological Diversity (CBD), the 193 parties to the Convention approved the Aichi Biodiversity Targets, including a goal to prevent extinctions (target 12). The 2015 United Nations 2030 Agenda for Sustainable Development includes Sustainable Development Goals (SDG) to halt biodiversity loss and prevent the extinction of threatened species (target 15.5). Although all of these commitments may not be fully met, they provide a fulcrum for applying pressure for the reordering of priorities. There are also efforts to mainstream biodiversity conservation into economic sectors (e.g., through forest certification [Karlsson‐Vinkhuyzen et al. 2017]) and to apply international mechanisms through such intergovernmental organizations as the CBD (e.g., for marine fisheries [Friedman et al. 2018]), which can significantly affect overall investment in conservation. Environmental and public health crises, such as the COVID‐19 pandemic, which have a component of interaction between biodiversity and human health and well‐being, should also stimulate interest in investment in conservation, including engagement with the public health donor community. The resources to save species do exist, although adequate funds are not at present allocated to conservation.

Besides the public sector, biodiversity conservation relies on significant private investments, some of which are available through economic mainstreaming of conservation (see above), impact investment (Shames & Scherr 2020), and carbon‐credit investment projects. More specifically, species conservation is replete with examples of new and expanded private sector resources, either financial or human effort, being procured for threatened species conservation that were not available to conservation prior to the commencement of conservation activities. Boosting the recognition of a species’ plight can trigger additional resources for that species, at least in the short to medium term. Many organizations have stepped up to add resources to conservation efforts for specific species or groups of species, including new organizations that bring entirely new resources, such as the Mohamed bin Zayed Species Conservation Fund, the International Union for Conservation of Nature Save Our Species Rapid Action Grants, and the National Geographic Society Recovery of Species on the Brink of Extinction program. Because the establishment of new organizations in some regions is often related to funding, species conservation is likely benefiting from an increased flow of funding.

Multilateral initiatives have also arisen, such as The Global Environment Facility (GEF). These organizations have not served merely to reallocate existing funds to their preferred target species, but have tapped into new sources of funding, thereby expanding the overall conservation funding pool. They have done this by raising public awareness of the conservation issues faced by their target species or group. Public involvement has in turn brought new, direct sources of funds from private donations and has applied pressure to governments via advocacy and citizen concerns to provide additional financial resources and to support actions benefiting threatened species. A recent example is the increasing commitment to insect conservation (Harvey et al. 2020).

In addition to resources directed to single species, conservation can take advantage of significant synergies, combinations of resources that may benefit not only multiple species, but also additional components of ecosystem functioning. Resources for conservation are not a zero‐sum game, where resources allocated to species B necessarily withdraw resources from species A. Conservation is inherently synergistic: efforts employed to save one species almost always benefit a number of others, multiplying efforts and achieving conservation for several species. Protecting or conserving a key site, habitat, landscape, or seascape for one threatened species almost always protects habitat for many others and improves overall ecosystem integrity and functionality. Conservation sometimes provides benefits by buffering climate effects, storing carbon, preventing erosion, and helping support many other ecological services. Conservation investment flows back into the global economy by creating jobs for forest guards, protected‐area managers, and a range of other professionals. When conservation includes sustainable wildlife tourism, it can provide significant economic and livelihood benefits for local communities. As an example, ecosystem services and other values of the 67 giant panda (*Ailuropoda melanoleuca*) reserves in China were valued at between US$2.6 and $6.9 billion per year in 2010 dollars (Wei et al. 2018). This value was 10–27 times greater than the cost of maintaining the reserves, representing a very positive benefit–cost ratio. Of course, not all threatened species may present such favorable economic scenarios, but in other cases natural areas have been shown to have positive benefits (Balmford et al. 2002).

Another important factor affecting conservationists’ ability to expand human and financial resources available for the prevention of species loss is time scale. The trajectory toward extinction for species is not usually measured in hours or days, but rather in years, decades, or even centuries (e.g., Schorger 1955; Fuller 2002). Once a threat to a species is recognized, prompt action from nimble small‐grant programs can activate near‐immediate resources that can slow the extinction trajectory for a species (Brooke et al. 2008) while allowing time for additional resources to be mobilized. In emergency situations, such as apparent rapidly impending species extinctions, new resources are frequently mobilized from novel sources to save species. There is often time available between recognition of a threat and potential extinction, time during which the threatened status of a species can be brought to public attention, funds can be raised, threats can be researched and addressed, guardians can be trained, and other resources can be made available and brought to bear. To save the most threatened species, it is not necessary to immediately expand conservation funding to the maximum prescribed (McCarthy et al. 2012). During the time required to successfully access resources, it is possible to make decisions on where to focus conservation efforts for maximum reduction in extinction avoidance. These decisions do not have to focus on which species should be allowed to go extinct, but rather on which to focus first, with the objective that no species would be allowed to be extinguished.

## Recoverability of All Species

Besides the idea that resources are overwhelmed, the concept of conservation triage suggests that some species cannot be recovered at all, not at any cost or benefit–cost ratio. Although there clearly are some cases in which a serious and adequately funded attempt at conservation may end with a species being conservation dependent, there are few cases where conservation action cannot reduce extinction risk and conservation dependency and considerable evidence that it is generally not true. The failure of a specific conservation effort does not support the idea that conservation should not have been attempted.

One condition often identified as a significant risk for extinction, which thereby places a species into the bottom category of triage (i.e., no resources should be expended), is small population size, as in the case of the vaquita (Cornwall 2018). Small population size alone, however, does not doom a species. There are numerous cases of species with very low population sizes that have rebounded. The Española giant tortoise (*Chelonoidis hoodensis*) had a population of only 15 individuals in 1974, but a strong captive breeding and reintroduction program has increased the population to well over 1500 individuals (Gibbs et al. 2014). The Lord Howe Island stick insect (*Dryococelus australis*) was presumed extinct for 80 years before its rediscovery in 2001 (Priddel et al. 2003), with a population of 24 individuals, and captive breeding now has produced a captive population of more than 14,500 individuals thanks to the efforts of the Melbourne Zoo (Australia) (Quintanilla 2017) and partners. The tree *Ramosmania rodriguesii* was down to a single individual, but now numbers in the hundreds (Magdalena 2015). There are numerous natural situations in which species had very low populations and then either recovered or persisted for a very long time. Many species of vertebrates on islands appear to have never occurred in large numbers, from perhaps 500 individuals up to a few thousand, yet these species have persisted for millennia (Walter 2004). Many additional examples are given in Appendices S1–S5. These examples show that natural recovery or conservation carried out on behalf of even very small populations can increase their population sizes. Therefore, it should not be assumed that species that have reached some arbitrary lower threshold are doomed to extinction.

With small populations, however, there are always concerns about the additional risks posed by restricted population size, notably, a perceived risk of extinction from genetic inbreeding (Franklin 1980). Although genetic inbreeding is indeed negatively correlated with fitness, its consequences can vary substantially. For example, in the case of the Morro Bay kangaroo rat (*Dipodomys heermanni morroensis*), low genetic diversity is historical (Matocq & Villablanca 2001). African cheetahs have low genetic diversity dating back to the end of the Pleistocene, 10,000 years ago (Menotti‐Raymond & O'Brien 1993). Heterozygosity can be maintained by endogenous mechanisms that raise it above values expected at random (Frasier et al. 2013; Nowicki et al. 2019). Although the conservation of intraspecific genetic diversity is a valuable conservation target, genetic diversity is not necessarily useful in predicting the fate of species (Farrington et al. 2019).

Regardless of the potential for inbreeding, animals with certain life‐history characteristics, such as long‐lived iteroparous species (e.g., bog turtle [*Glyptemys muhlenbergii*]), can persist at low populations (Shoemaker et al. 2013), below the limits suggested by the 50/500 rule (Franklin 1980), for example. (Although now rarely seen in peer‐reviewed literature, the 50/500 rule is still at times invoked by field practitioners, especially where resources are few and access to current literature is financially limited.) The rule only addresses the question of what the effects on the species would be in the long term if nothing unusual happens (Caughley 1994). Even for shorter‐lived species, the long‐term trajectory can be affected positively by conservation action, such as directed genetic mixing. Small population is a risk factor for species on several fronts, but does not itself provide a hard threshold below which a species should be considered unrecoverable, as suggested by the 50/500 rule. Given sufficient resiliency and conservation attention, even tiny populations of species have been shown to persist.

New tools have been developed that move more species from assessment to action via planning more quickly (Lees & Gibson 2019). In terms of implementation, ex situ management (seed banking, cryopreservation, captive breeding, maintenance of assurance colonies, and head starting to support population augmentation) is an effective technique for extinction prevention. Practitioners are becoming increasingly skilled at these and other practical conservation techniques, such as the eradication or control of invasive species on islands. The political framework for species conservation has also improved with the advent of initiatives such as the UN Sustainable Development Goals (which call for the reduction of habitat degradation and loss of biodiversity), the Convention on Biological Diversity, the Convention on International Trade in Endangered Species of Wild Fauna and Flora, and the Convention on the Conservation of Migratory Species of Wild Animals; funding mechanisms such as The Global Environment Facility and the Critical Ecosystems Partnership Fund; lending institutions creating policies to prevent the most negative environmental outcomes from infrastructure projects their loans support; and the economic mainstreaming of biodiversity. These policy and funding developments are further enabling species conservation despite threats that are often increasing.

This means that, with an explicit goal of avoiding extinctions, such extinctions should be easier to prevent as time advances. This is not to say that the variety and intensity of threats to species are decreasing. New threats—arising from human population growth, overconsumption, greenhouse‐gas emissions, rapid transportation spreading invasive species and diseases, increased overexploitation and trade, and many other sources—place increasing pressure on species. Improving organizational structures and more advanced knowledge, however, can and do make species conservation more effective.

## No Extinctions as an Explicit Goal of Prioritization Systems

Because conservation triage is not necessary or ethical, it should be avoided. Although current systems of conservation resource prioritization may not explicitly allow for species to be extinguished, the practical effect of these systems is to allow extinction. Although a prioritization system may present policy makers with a range of options that include conservation of all species, those policy makers can choose to eliminate projects that have high cost for low probability of success. Triage therefore may serve to increase success rate and thus be a tool to satisfy politicians and donors, but it will inevitably lead to reaching for the low‐hanging fruits. Zero extinctions is not an emergent property of systems of conservation resource prioritization. Because there is no rationalization for allowing extinctions, this must be an explicit goal of the prioritization system.

One of the first steps in a prioritization system, such as that of Joseph et al. (2009), is to set the objectives for the prioritization exercise. It is at this stage that an explicit goal of allowing no extinctions should be included, not just an objective of conserving the greatest possible number of unique species. Without this goal, the prioritization system becomes a triage system because species assigned low values based on the economics of their recovery can be allocated no resources for their recovery.

## Conclusion

Although setting priorities with predetermined systems would seem to create efficiency in a resource‐limited system, global resources for species conservation are less limited and more expandable than many believe. There is also a moral dilemma in selecting among species that cannot be solved through the application of science.

Although resources to conserve all threatened species are not allocated in sufficient quantity presently or distributed where they are most required, the world's vast economic and intellectual resources are more than adequate to meet conservation needs, especially if one designs disbursement mechanisms, such as rapid‐action small grants, that are appropriate to the scale of field projects. In fact, because many species conservation activities are carried out by small nongovernmental organizations working in remote areas and with limited capacity, small‐grant mechanisms can be one of the most effective ways of providing direct support for species. Indeed, if more funds could be allocated through such mechanisms, perhaps the total amount needed to achieve species conservation would be far less than some of the estimates. Although the political or philanthropic will to allocate the needed resources may not yet exist for a doubling, tripling, or 10‐fold increase in conservation funding, those resources will become available if the propensity to grant them can be cultivated. The COVID‐19 pandemic complicates matters, but offers opportunities to rethink and optimize economic resources for human development and biodiversity conservation.

As targets of zero extinction and prevention of endangerment become more widely acknowledged in conservation policy (as through the CBD and SDGs), the political will to increase conservation funding is likely to increase (Bolam et al. 2020). Especially if species conservation can be bolstered by considering the species’ intrinsic value and benefits to people (economic, cultural, and health), the ultimate cost will not be beyond what can be borne by the global system and should be considered an investment in that system rather than an expense. In fact, existing small‐funding systems with agile and quick responses have shown that small amounts of money can make significant differences to the conservation status of species.

We should be asking how we can best address the conservation needs of species, not whether the needs can be addressed. We call on conservationists to work together to understand, develop, and invest in the resources needed to maintain and recover all species, not just those for which the task may seem less challenging. Rather than settling for a flawed system of triage, striving to recover all species and prevent as many as possible from becoming threatened keeps all of the pieces of biodiversity together and follows Aldo Leopold's dictum, “To keep every cog and wheel is the first precaution of intelligent tinkering” (Leopold 1966). Extinction is irreversible, and should not—and need not—be acceptable. The explicit goal of zero extinction is a key paradigm in species conservation, one which should not and cannot be dismissed.

## Supporting information

Additional information is available online in the Supporting Information section at the end of the online article. The authors are solely responsible for the content and functionality of these materials. Queries (other than absence of the material) should be directed to the corresponding author.
