# Supplementary material for: Conservation resource allocation, small population resiliency, and the fallacy of conservation triage
Source: Conserv Biol. 2021 May 4;35(5):1388–95. doi: 10.1111/cobi.13696 (PMC8518633; doi:10.1111/cobi.13696)
Supplement: Supplementary file 1 — Additional information is available online in the Supporting Information section at the end of the online article. The authors are solely responsible for the content and functionality of these materials. Queries (other than absence of the material) should be directed to the corresponding author. [file COBI-35-1388-s001.docx]

**Supporting Information**

**Appendix S1. Ten examples of bird species with populations that have dipped below 100 but have since increased** as a result of conservation action. Historic population lows and recent highs are given with year in parentheses. Population numbers include both wild and captive. Numbers are from the BirdLife International Data Zone (accessed August 2018) unless other reference is given.

| **Species** | **Minimum population (year)** | **Most recent population (year)** | **References** |
| --- | --- | --- | --- |
| Pink pigeon *Nesoenas mayeri* | 10 (1990) | 370-380 (2013) |  |
| Whooping crane *Grus americana* | 15 (1938) | 621 (2014) |  |
| Bermuda petrel *Pterodroma cahow* | 36 (1951) | 250 (2005) |  |
| Asian crested ibis *Nipponia nippon* | 12 (1981) | 2,600 (2019) | Feng et al. 2019 |
| California condor *Gymnogyps californianus* | 22 (1981) | 463 (2017) | USFWS 2017 |
| Mauritius kestrel *Falco punctatus* | 4 (1974) | 350-500 (2013) |  |
| Puerto Rican amazon *Amazona vittata* | 13 (1975) | 505 (2013) |  |
| Millerbird *Acrocephalus familiaris* | 50 (1990) | 1,057 (2014) |  |
| Bali myna *Leucopsar rothschildi* | 15 (1990) | 1,115 (2008) |  |
| Pale-headed brush-finch *Atlapetes pallidiceps* | 12 (1998) | 226 (2009) | Agreda et al. 1999 |

**Appendix S2. Ten examples of mammal species with populations that have dipped below 100 but have since increased** as a result of conservation action. Historic population lows and recent highs are given with year in parentheses. All population numbers include both wild and captive, if any. Numbers are from the IUCN Red List of Threatened Species (accessed January-March 2019) http://www.iucnredlist.org unless other reference is given.

| **Species** | **Minimum population (year)** | **Most recent population (year)** | **References** |
| --- | --- | --- | --- |
| Gilbert’s potoroo *Potorous gilbertii* | 8 (1999) | 70 (2011) | Vaughan-Higgins et al. 2011 |
| Northern hairy-nosed wombat *Lasiorhinus krefftii* | 35 (1980s) | 250 (2016) | Eastwood 2003, Horsup 2004, Horsup 2018 |
| Rodrigues fruit bat *Pteropus rodricensis* | 70 (1979) | 20,000 (2017) |  |
| European bison *Bison bonasus* | 54 (1920s) | 3,200 (2008) |  |
| Arabian oryx *Oryx leucoryx* | 11 (1978) | 6,000 – 7,000 (2016) | Henderson et al. 1974, International Union for Conservation of Nature 2017 |
| Pere David’s deer *Elaphurus davidianus* | 12 (1890s) | Several thousand (2015) | Jiang et al. 2000 |
| Przewalski’s horse *Equus ferus* | 12 (1902) | 1,988 (2014) |  |
| Southern white rhinoceros *Ceratotherium s. simum* | 20 (1900) | 20,000 (2010) | ‘t Sas-Rolfes 2011 |
| Black-footed ferret *Mustela nigripes* | 18 (1987) | 698 (2013) | Black-footed Ferret Connections 2019, USFWS 2013 |
| Golden-headed langur *Trachypithecus poliocephalus* | 50 (2000) | Low 50s (2015) | Leonard et al. 2017 |

**Appendix S3. Examples of reptile species with populations that have dipped below 100 but have since increased** as a result of conservation action. Historic population lows and recent highs are given with year in parentheses. Population numbers include both wild and captive. Numbers are from Gibbs et al. 2014, Cayot et al. 2017, TCC [Turtle Conservation Coalition] 2011, or TCC [Turtle Conservation Coalition] 2018, unless other reference is given.

| **Species** | **Minimum population (year)** | **Most recent population (year)** | **References** |
| --- | --- | --- | --- |
| Western swamp turtle *Pseudemydura umbrina* | 30 (1987) | 300 (2018) |  |
| Myanmar roofed turtle *Batagur trivittata* | 10 (2002) | 1,000 (2018) |  |
| Yunnan box turtle *Cuora yunnanensis* | 3 (2006) | 70 (2018) |  |
| Española giant tortoise *Chelonoidis hoodensis* | 15 (1974) | 1,500 (2014) |  |
| Pinzón giant tortoise *Chelonoidis duncanensis* | < 100 (1990) | 500 (2017) |  |
| Grand Cayman blue iguana *Cyclura lewisi* | <120 (2002) | 443 (2012) | Burton 2002, Tandora Grant, International Rock Iguana Studbook keeper (pers. comm.) |
| Jamaican iguana *Cyclura collei* | 50 (1991) | 399 (2018) | Tandora Grant, International Rock Iguana Studbook keeper (pers. comm.) |

**Appendix S4. Examples of fish species with populations that have dipped below 100 but have since increased** as a result of conservation action. Historic population lows and recent highs are given with year in parentheses. Population numbers include both wild and captive except where stated.

| **Species** | **Minimum population (year)** | **Most Recent Population (year)** | **References** |
| --- | --- | --- | --- |
| Azraq Toothcarp *Aphanius sirhani* | Almost zero (1992) | Estimated at 100,000 (2016) | Nashat Hamidan and Jörg Freyhof (pers. comm.) |
| Devil's Hole Pupfish *Cyprinodon diabolis* | 35 (2013) | Wild 170 (2019)  Captive 250 (2021) | Kevin Wilson and Jenny Gumm (pers. comm.) |
| Big Bend Gambusia *Gambusia gaigei* | 3 (1956) | Several thousand but fewer than 10,000  (2012) | NatureServe, 2013; Jenny Gumm (pers. comm.) |
| Adriatic Grayling *Thymallus aeliani* | 50-100 (2010) | Thousands (2018) | Cesare Puzzi and Jörg Freyhof (pers. comm.) |
| Thermal rudd *Scardinius racovitzai* | < 100 (2014) | Several hundred (2018) | Müller et al. 2018, Jörg Freyhof and Tamàs Müller (pers. comm.) |

**Appendix S5. Examples of plant species with populations have that dipped below 100 but have since increased** as a result of conservation action. Historic population lows are given with year in parentheses. All population numbers include both wild and cultivated (nursery/garden/introduced). Numbers are from the IUCN Red List of Threatened Species (accessed January-March 2019) http://www.iucnredlist.org unless other reference is given.

| **Species** | **Minimum population (year)** | **Most Recent Population (year)** | **References** |
| --- | --- | --- | --- |
| *Pennantia baylisiana* | 1 (1945) | >1,600 (2010) | Platt 2010 |
| Ōlulu *Brighamia insignis* | <40 (1994) | Hundreds (2008) | USFWS 2008 |
| *Erica verticillata* | 8 (1980s) | A few hundred (2018) | Kenilworth Racecourse Conservation Area 2019 |
| Toromiro *Sophora toromiro* | 1 (1958) | Several thousand (2015) | Maunder et al. 2000, Portal del Plan Nacional de Conservación del Toromiro 2019 |
| *Cyclobalanopsis (Quercus) sichourensis* | 5 (2007) | Thousands (2016) | Xia et al. 2016 |
| *Ramosmania rodriguesii* | 1 (1986) | Hundreds (2015) | Magdalena 2015, Owens 1993 |
| *Magnolia sinica* | 50 (2005) | 5,000-6,000 (2018) |  |
| *Trochetiopsis ebenus* | 2 (1980) | Thousands (2015) |  |
| *Turbina inopinata* | 24 (before 2007) | 81 (2007) |  |

Literature Cited in Appendices

Agreda, A, Krabbe, N, Rodríguez, O. 1999. Pale-headed Brush Finch *Atlapetes pallidiceps* is not extinct. Cotinga **11**: 50–54.

Black-footed Ferret Connections (or is the author blackfootedferret.org?). 2020. History of the black-footed ferret, rediscovery and second chances. Black-footed Ferret Connections. Available from <https://blackfootedferret.org/history/> (accessed January 2021).

Burton, FJ. 2002. Grand Cayman blue iguanas in the wild: A survey of the population status of *Cyclura nubila lewisi*. Blue Iguana Conservation Project working report, 2002. National Trust for the Cayman Islands, South Sound, Cayman Islands

Cayot, LJ, Gibbs, JP, Tapia, W, Caccone, A. 2019. *Chelonoidis duncanensis*. The IUCN red list of threatened species 2017. International Union for Conservation of Nature, Gland, Switzerland. Available from  <https://dx.doi.org/10.2305/IUCN.UK.2017-3.RLTS.T9021A3149054.en>.

Eastwood, K. 2003. Saving the northern hairy-nosed wombat. Australian Geographic **October–December**, 72.

Feng, S, et al. 2019. The genomic footprints of the fall and recovery of the Crested Ibis. Current Biology **29**: 340–349.

Henderson, DS. 1974 Were they the last Arabian oryx? Oryx **12**: 347-350.

Horsup, A. 2004. Recovery plan for the northern hairy-nosed wombat (*Lasiorhinus krefftii*)*.* Report. Department of Environment and Heritage, Canberra, Australia.

Horsup, A. 2018. Northern hairy-nosed wombat. State of Queensland, Department of Environment and Heritage Protection, Brisbane. Available from <https://environment.des.qld.gov.au/wildlife/threatened-species/endangered/northern_hairynosed_wombat/#how_many_species_of_wombats> (accessed January 2021).

International Union for Conservation of Nature (IUCN). 2017. *Oryx leucoryx*. The IUCN red list of threatened species. Version 2020-1. IUCN, Gland, Switzerland (accessed May 2020).

Jiang, Z, Yu, C, Feng, Z, Zhang, L, Xia, J, Ding, Y, Lindsay, N. 2000. Reintroduction and recovery of Père David's deer in China. Wildlife Society Bulletin **28**: 681-687.

Kenilworth Racecourse Conservation Area. No year. The Story of the Erica Verticillata. Kenilworth Racecourse Conservation Area, Kenilworth, South Africa. Available from http://krca.co.za/extinct_erica_verticillata (accessed January 2021).

Leonard, N, Passaro, RJ, Schrudde, D, Stenke, R, Thuc, PD, Raffel, M. 2017. Golden-headed or Cat Ba Langur *Trachypithecus poliocephalus* (Trouessart, 1911). Pages 59-60 in Schwitzer, C, Mittermeier, RA, Rylands, AB, Chiozza, F, Williamson, EA, Macfie, EJ, Wallis, J, Cotton, A, editors. Primates in peril: The world’s 25 most endangered primates 2016-2018. IUCN SSC Primate Specialist Group, International Primatological Society, Conservation International and Bristol Zoological Society.

Maunder, M, Culham, A, Alden, B, Zizka, G, Orliac, C, Lobin, W, Bordeu, A, Ramirez, JM, Glissmann‐Gough, S. 2000. Conservation of the toromiro tree: case study in the management of a plant extinct in the wild. Conservation Biology **14**: 1341-1350.

Müller, T, Bernáth, G, Horváth, Á, Várkonyi, L, Grigoras, G, Gagiu, A, Urbányi, B, Zarski, D, Freyhof, J, Cameron, T. 2018. Artificial propagation of the endangered Rumanian endemic warm water rudd (*Scardinius racovitzai* Müller 1958, Cyprinidae, Cypriniformes) for conservation needs. Egyptian Journal of Aquatic Science 44: 245-249

NatureServe. 2013. *Gambusia gaigei*. The IUCN Red List of Threatened Species 2013: e.T8890A18229201. DOI: 10.2305/IUCN.UK.2013-1.RLTS.T8890A18229201.en

Owens, SJ, Jackson, A, Maunder, M, Rudall, P, Johnson, MA. 1993. The breeding system of *Ramosmania heterophylla*–dioecy or heterostyly? Botanical Journal of the Linnean Society **113**: 77-86.

Platt, J. 2010. World’s rarest tree gets some help. Scientific American blog: <https://blogs.scientificamerican.com/extinction-countdown/worlds-rarest-tree-gets-some-help/> (accessed January 2021).

Portal del Plan Nacional de Conservación del Toromiro. 2017. Plan nacional de conservación del Toromiro. Corporación Nacional Forestal, Santiago, Chile. Available from http://plantoromiro.org (accessed January 2021).

‘t Sas-Rolfes, M. 2011. Saving African rhinos: a market success story. Property and Environment Research Center, Bozeman, Montana.

TCC [Turtle Conservation Coalition]. 2011. Turtles in trouble: the world’s 25+ most endangered tortoises and freshwater turtles—2011. IUCN/SSC Tortoise and Freshwater Turtle Specialist Group, Turtle Conservation Fund, Turtle Survival Alliance, Turtle Conservancy, Chelonian Research Foundation, Conservation International, Wildlife Conservation Society, and San Diego Zoo Global.

TCC [Turtle Conservation Coalition]. 2018. Turtles in trouble: the world’s 25+ most endangered tortoises and freshwater turtles—2018. IUCN SSC Tortoise and Freshwater Turtle Specialist Group, Turtle Conservancy, Turtle Survival Alliance, Turtle Conservation Fund, Chelonian Research Foundation, Conservation International, Wildlife Conservation Society, and Global Wildlife Conservation.

U.S. Fish and Wildlife Service (USFWS). 2008. *Brighamia insignis* (Olulu), 5-Year review: summary and evaluation. USFWS Pacific Islands Fish and Wildlife Office, Honolulu, Hawaii, U.S.A.

U.S. Fish and Wildlife Service. 2017. California Condor Recovery Program 2017 annual population status. California Condor Recovery Program, U.S. Fish and Wildlife Service Pacific Southwest Regional Office, Sacramento, California, U.S.A.

U.S. Fish and Wildlife Service. 2013. Recovery plan for the black-footed ferret (*Mustela nigripes*), second revision. U.S. Fish and Wildlife Service Region 6 Office, Lakewood, Colorado, U.S.A.

Vaughan-Higgins, R, Buller, N, Friend, JA, Robertson, I, Monaghan, CL, Fenwick, S, Warren, K. 2011. Balanoposthitis, dyspareunia, and *Treponema* in the Critically Endangered Gilbert’s potoroo (*Potorous gilbertii*). Journal of Wildlife Diseases **47**: 1019–1025.

Xia, K, Fan, L, Sun, W, Chen, W. 2016. Conservation and fruit biology of Sichou oak (*Quercus sichourensis*, Fagaceae)—a critically endangered species in China. Plant Diversity **38**: 233-237.
